# Preparation, Characterization, and Performance Control of Nanographitic Films

**DOI:** 10.3390/nano9040628

**Published:** 2019-04-17

**Authors:** Shumin Chen, Qiang Jiang, Yong Chen, Lulu Feng, Di Wu

**Affiliations:** 1 School of Mathematics and physics, Jingchu University of Technology, Jingmen 448000, China; chenyong3089@126.com (Y.C.); Fll85013296@wust.edu.cn (L.F.); q18371166703@163.com (D.W.); 2 State Key Lab Precis Measurement Technology & Instrument, Tsinghua University, Beijing 100084, China; jiangqiangjq@126.com

**Keywords:** nanographitic films, PECVD, growth mechanism, optoelectronic property

## Abstract

Using methane as a carbon source, low-dimensional carbon nanomaterials were obtained in this work. The films were deposited directly on glass substrates by radio frequency plasma-enhanced chemical vapor deposition (RF-PECVD). The configuration and compositions of this nanographite films were identified by X-ray photoelectron spectroscopy (XPS) as carbon in sp^2^ bonding form. Raman spectral characterization verified the configuration of the films to be hexatomic ring of carbon atoms. As a result, they were found to be nanographite films (NGFs). Also, the atomic force microscopy (AFM) topography and Raman spectra of different areas demonstrated the diversity of the films at the nano scale. The high light-transmitting and electron mobility indicated that the NGFs possessed excellent optic-electronic properties and could be used as good photoelectrical function materials. Furthermore, the physical and chemical growth mechanism of NGFs were analyzed by PECVD. NGFs could be obtained in a controlled process by modulating the growth conditions. In this work, the complicated transfer process commonly used for optoelectronic devices could be avoided. Also, by growing the films directly on a glass substrate, the quality degradation of the film was not a problem. This work can further promote the development of next-generation electronic or optoelectronic function materials, especially for their application in transparent conductive electrode fields.

## 1. Introduction

As one of the most intensively investigated traditional materials, carbon has a history of thousands of years. Carbon is one of the most abundant elements in the earth’s crust. It is found in many allotropes and forms. With the continued scientific exploration of carbon materials, graphite, diamond, fullerene, carbon nanotube, and graphene were discovered successively in the 20th century during 50 years [1,2,3,4]. They have been extensively used in many fields. The 2010 Nobel Prize for physics was awarded to Andre Geim and Konstantin Novoselov for “ground-breaking experiments regarding the two-dimensional material graphene” [5]. This breakthrough stirred up scientists’ enthusiasm for research. A wide variety of carbon materials and related preparation methods were reported [4,5,6]. Graphene film, a unique two-dimensional (2D) monoatomic planar membrane of carbon, has emerged as a revolutionary breakthrough in material technology. Owing to their excellent transparency, electroconductivity and air stability, graphene and its related low-dimensional carbon nanomaterials have been used as transparent conductive electrodes and electron transport layer and buffer layer for photoelectric devices [7,8,9,10,11,12,13]. As reported in previous studies, carbon-related low-dimensional nanomaterials possess outstanding characteristics. They showed enough strength and superior mechanical performance, excellent physical and chemical stability, high thermal conductivity, high specific surface area, excellent optical property, and good conductivity. These versatilities are very promising in a wide range of application domains, such as machinery and electronics, aerospace, photoelectric devices, energy conservation, and bio-pharmaceuticals, which made them become the rapidly rising star on the horizon of materials science. 

These significant progresses have greatly promoted the fabrication and application of low-dimensional carbon nanomaterials. However, there are lots of important challenges that limit the practical application of low-dimensional carbon nanomaterials [14,15,16]. At present, the obtained low-dimensional carbon nanomaterials films and their related materials need to be separated from metal substrates and transferred to insulating substrates for further electronic processing [16]. It is a tedious and lengthy process, which demands higher operating costs, and the materials present poor performance. So, growing low-dimensional carbon nanomaterials directly on insulating substrates such as Si/SiO_2_ or glass is helpful to overcome the quality degradation and additional defects caused by the transfer process [15]. However, growing graphene-related materials directly on a Si or glass substrate is still a great challenge. In addition, low-temperature carbon film growth is attractive to reduce the costs and may enable the direct growth of carbon materials on flexible polymer-based substrates. However, the currently typical temperature required for chemical vapor deposition (CVD) growth of carbon-related materials is 800 °C–1000 °C [17,18,19,20], which is still too high. To address these problems, plasma-enhanced chemical vapor deposition (PECVD) was chosen for the preparation. PECVD has been widely used to produce carbon-related materials because of its advantages [8,9,10]. Compared to other CVD methods, PECVD possesses advantages such as lower reaction temperature, higher growth selectivity, purer atmosphere, and easiness in realizing large growth areas of films [5,8,9,10]. In this work, nanographitic films (NGFs) were deposited directly on glass substrates without a further transfer process. Radio frequency (RF)-PECVD was used to synthesize NGFs under 300 °C. This temperature is much lower than the temperature required for the growth of carbon films by CVD. Meanwhile, the sole source gas that was used was methane without hydrogen, which is rare in most of the carbon-related materials synthetic procedures using CVD. Furthermore, the structure and the optical and electronic properties of the films were characterized by Raman spectroscopy, ultraviolet–visible spectrophotometry, and hall effect measurement system, respectively.

## 2. Materials and Methods

The NGFs were synthesized by a 450 high-vacuum RF-PECVD system (manufactured by Xin Lan Tian vacuum technology Co., LTD, Shenyang, China). Glass (1–2 mm in thickness, CAT.NO.7101) was chosen as a substrate, and methane was used as the precursor. The glass substrate was washed with acetone, alcohol, and deionized water three times, separately. Then, the cleaned substrates were placed onto the chassis. The air pressure of the chamber was kept at 10^−4^. A gaseous mixture containing a certain ratio of carbon source (methane) and assistant gas (argon) was imported into the chamber. Then, the chassis was gradually heated to 300 °C. The radio frequency power was then turned on, and the power was set to 200 W. Thus, plasma was generated by glow discharge. After the growth process, the RF power was shut off, and the chamber was cooled down naturally to room temperature. For a comparative study, the growth parameters were carefully controlled. A variety of variables were modulated in this work, including vacuum pressure, substrate temperature, radio-frequency power, gas flow, deposition time, and space between the polar plates. The optimum experimental parameters are listed in Table 1. Finally, NGFs with controlled morphologies and structures formed on the glass.

The surface morphology and thickness of the films were characterized by scanning electron microscope (SEM), transmission electron microscope (TEM), and atomic force microscopy (AFM). The configuration and compositions of these NGFs were characterized by X-ray photoelectron spectroscopy (XPS), and their bonding forms and properties were obtained by Raman spectroscopy. The optical properties of the films were characterized by ultraviolet–visible spectrophotometry. The JSM-6700F SEM (manufactured by JEOL Ltd. from Japan), with a limit resolution up to 50 À, could reach a magnification of 50–100,000 times, and the accelerating voltage could be modulated in the range of 1–40 KV. The Cypher S AFM was manufactured by Asylum Research from America. The INVIA confocal Raman spectrometer (manufactured by Renishaw PLC from England) was used with a typical laser wavelength at 633 nm. The laser beam was focused on the sample with a spot size of 2.0–3.0 µm in diameter. The Persee TU-1901 double-beam mode UV-spectrophotometer was produced by the Beijing Purkinje General Instrument Limited company. The range of spectrum was 300–850 nm, and the spectral bandwidth was 1 nm. The electrical properties, such as carrier concentration, carrier mobility, and resistivity of the films transferred to the SiO_2_/Si substrate were tested by a Crosstech HMS300 hall effect test system, which was manufactured by ACCEAT OPTICAL Company from England.

## 3. Results and Discussion

### 3.1. Morphology Characteristics of the NGFs

**Figure 1:**
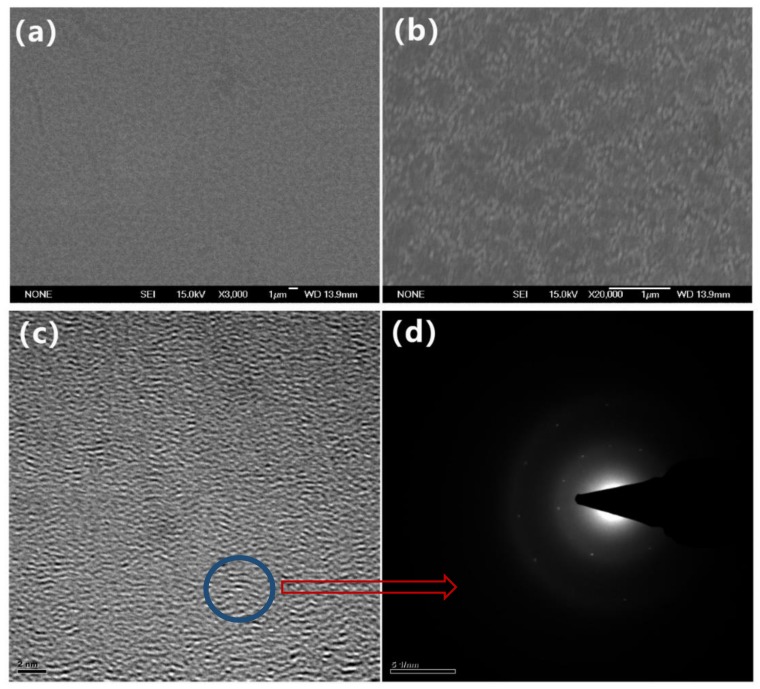
(**a**) Surface topography of NGFs by SEM with magnification of 3000x; (**b**) surface topography of NGFs by SEM with magnification of 20,000x; (**c**) morphologies of NGFs by TEM; (**d**) transmission electronic diffraction pattern of the area indicated in (**c**).

**Figure 2:**
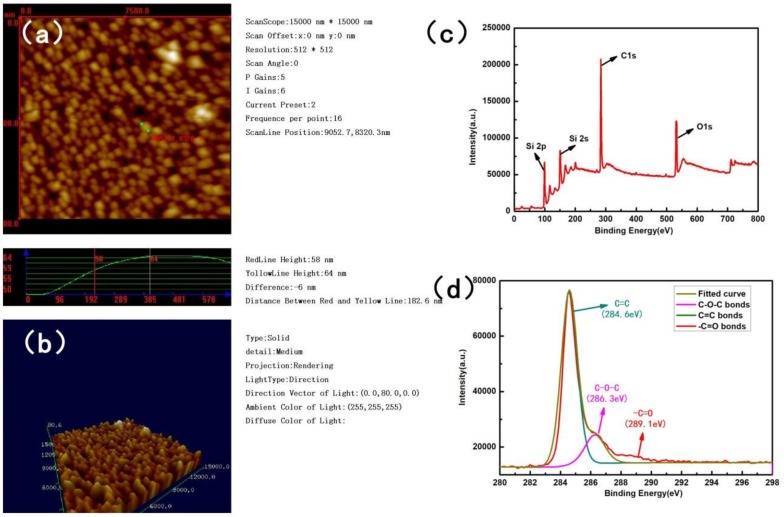
(**a**) 2D AFM images of the films; (**b**) 3D AFM topography of the films; (**c**) XPS wide surface survey of the NGFs; (**d**) narrow scans for C1s.

As shown in Figure 1, the morphologies of the samples obtained by RF-PECVD were observed by SEM. A typical sample was selected with the following preparation conditions: 50 Pa, 200 W, 2:50 sccm, 5 cm, 60 min, and 300 °C. Figure 1a shows the surface topography with magnification of 3000x. As we can see, layers of uneven films with different thicknesses and sizes were distributed on the surface of the glass substrate. As shown by the scale bar, the maximum size of these films could reach 1 µm. Figure 1b presents a larger version of the films, with a magnification of 20,000x. It shows that there were layers of compact film with fluctuating sizes on the nanoscale on the substrate. Figure 1c,d clearly show the atoms’ crystal lattice of the NGFs by TEM. It presents that the morphology of this region had features of lattice gradient and interleaving structure. The selected area in Figure 1c was analyzed to observe the electron diffraction pattern. In the transmission electronic diffraction pattern of Figure 1d, a series of concentric circles with less obvious diffraction spots of regular hexagon can be seen. It demonstrates a polycrystalline structure of the NGFs, which is the character of carbon materials. The morphology at the nanometer scale can be clearly seen. On the other hand, it is evident that these interfaces were clean without any other reaction products. In order to further prove the thickness of the films, the surface roughness was studied by AFM in terms of root-mean-square roughness. Figure 2a,b presents the AFM topography of the films in 2D and 3D images. It shows a densely packed mass of atoms going ups and down, with an average height of 50–80nm. Two representative points were selected and were marked in green, as shown in Figure 2a. The height difference was measured and calculated to be 6 nm. The 3D image in Figure 2a vividly displays the uneven surface of atomic dimensions. The deposition sequence, sectional structure, and grain size can be seen clearly. These results are in accordance with the SEM images and are useful for the analysis of the film growing mechanism.

### 3.2. Structural and Photoelectric Properties of the NGFs

The components and chemical states characterization of the NGFs previously analyzed by SEM is shown in Figure 2c,d. Figure 2c shows the XPS wide surface survey of the NGFs on the glass substrate. As shown, the strongest peak at 284–285 eV is characteristic of C1s, indicating that the major ingredient of NGFs was carbon. The peak of O1s at 529–537 eV with weak intensity was attributed to absorbed oxygen from the atmosphere and the glass substrate [20]. The very tiny peaks of Si2p and Si2s at 96–102 eV and 146–155 eV were attribute to SiO_2_ and its related compound from the glass substrate. Figure 2d exhibits the narrow scans for C1s. The C1s curve was deconvolved by using a standard multiple Lorentzian curves fitting, presented with different colors. As shown in Figure 2d, the line in army green located at 284.6 eV occupies a dominant position. It represents the peak of carbon–carbon double bounds. This is a convincing evidence that the carbon atoms with the basic six-membered ring structure of graphite were present, rather than other allotropes of carbon. The small pink peak at 286.3 eV was identified as the C–O–C bond [21]. The little peak in red at 289.1 eV could be featured as the O–C=O bond. These results further demonstrated the diverse structure of the NGFs.

**Figure 3:**
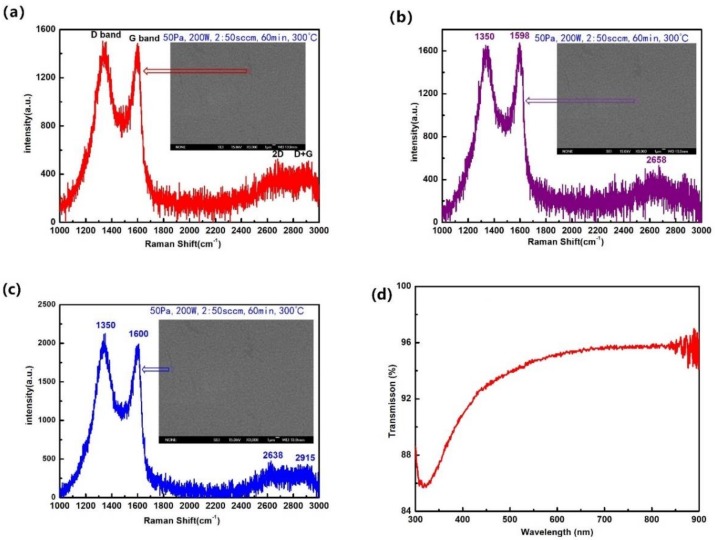
(**a**) Raman spectra of NGFs at the position marked by the red arrow. (**b**) Raman spectra of NGFs at the position marked by the purple arrow; (**c**) Raman spectra of NGFs at the position marked by the blue arrow; (**d**) transmittance of NGFs.

The structures and crystallinity quality of the films were studied by Raman spectroscopy. Figure 3a–c shows the Raman spectra of different points on the same sample, corresponding to the morphology shown in Figure 1 (these illustrations exhibit the specific position accordingly). A feature common to all of them is that there are four peaks named “D peak”, “G peak”, “2D peak”, “D + G peak”. The peak at about 1350 cm^−1^ was called “D band”. The D peak was due to the breathing modes of sp^2^ atoms in rings. This is a common defect-induced Raman feature of disordered graphitic carbon or is due to carbon at the edge of the film, which requires a defect for its activation [22,23,24,25,26]. The peak near 1595 cm^−1^ was due to bond stretching of sp^2^ atoms in both rings and chains, and is also known as G peak [23,24,27]. It is known to associate with the doubly degenerate phonon mode at the Brillouin zone center and indicated that the films were dominated by the sp^2^ sites of carbon. The “2D peak” centered at about 2650 cm^−1^ which originated from a second-order Raman process, is a typical peak of graphite [25,26]. The low intensity of the 2D peak and small I_2D_/I_G_ indicated a thick layer of nanographite. The very tiny peak at about 2950 cm^−1^ was a combination of the D and G peaks [24]. The thickness and quality of the films could be calculated by fitting the ratio of the intensity of the 2D peak to that of the G peak (I_2D_/I_G_). The full width at half maximum (FWHM) of the D peak, the G peak was influenced by the size and defects of crystals [23,24,25,26]. The linewidth of the D and G peaks and the Raman intensity ratio of the 2D and G peaks (I_2D_/I_G_) is shown in Table 2. As shown, the area marked by the red arrow in Figure 3a exhibited a less obvious 2D band. The broader and slightly upshifted 2D band as well as the reduction of I_2D_/I_G_ indicated an increase of the atom layers. So, the layer of film in the position marked by the purple arrow in Figure 3b was thinner than the layer of film in the red arrow. However, the 2D peak in Figure 3b is quite different from that of bulk graphite that consists of two components [27]. It can be seen that the D peak in Figure 3c was stronger and broader than the G peak, indicating that in this point (the area marked by the blue arrow), the film exhibited more defects and worse crystallization. It can be concluded that this point was on the edge, that is, at the junction between two six-membered rings. All these Raman spectrum results are consistent with the SEM graph shown in the nearby illustration. This is a powerful confirmation that there were ebbs and flows on the surface of the film, as seen in the AFM images in Figure 2. It is the imperfection and inhomogeneity of the films that caused differences of configuration in different areas. On the other hand, the positions and strength of the picks were slightly different. This further proved the nonuniformity of the films. In conclusion, the films were nanographite films with irregular surfaces. These results are in accordance with the SEM observations.

To explore the optical characteristics of the NGFs, the absolutely transmittance was measured and is shown in Figure 3d. From the spectra, it can be seen that the obtained NGFs had a very high transparency in visible wavelengths. Using ultraviolet light (460–300 nm), the transmissivity of NQFs declined significantly. The transmittance increased exponentially from 86% to 94%. This indicated that light in the UV region was absorbed more easily by the NGFs. In general, NGFs displayed excellent transmittance from 460 to 800 nm, close to 95–96%. This is very favorable for NGFs to be used as window layers in photoelectric devices. The electric properties of the NGFs, such as resistivity, mobility, and bulk concentration of the films, were measured by the Hall effect test system under 300 K. The average thickness of the film was about 200nm. Their carrier bulk concentrations could reach to 8.92 × 10^20^ cm^−3^. The NGFs exhibited n-type semiconductor characteristics with low resistance (1.89 × 10^−5^ Ω·cm) and relatively high mobility (654.8 cm^2^/V·S). Such outstanding carrier transporting performance make them very as the electron transport materials with capacious foreground. So, the NGFs could be successfully applied in photoelectric devices, especially as a transparent conductive layer [5,6,7,8,9,10].

### 3.3. Growing Mechanism of NGFs on a Glass Substrate

**Figure 4:**
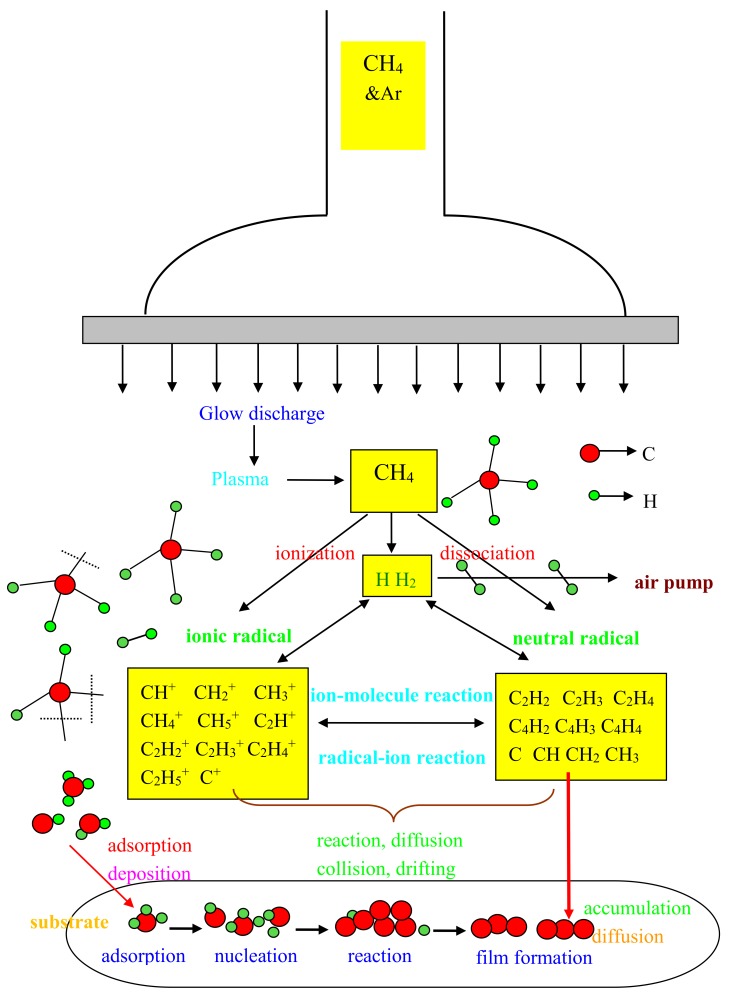
Detailed process of growing NGFs by RF-plasma-enhanced chemical vapor deposition (PECVD).

In order to grow the NGFs more controllably, their growth mechanism was researched intensively by conducting a series of experiments. By adjusting the deposition parameters, NGFs with different morphologies and structures were deposited on a glass substrate. To understand these complex processes, the detailed growth process of NGFs analyzed by RF-PECVD is shown in Figure 4. Considering that it is one of the cheapest small-molecule gases containing the carbon element and with a relatively lower pyrolysis temperature, methane was chosen as the carbon source. The deposition of NGFs could be divided into several processes: first, the carbon source of methane was highly activated and decomposed by plasma. The RF-PECVD equipment offered a sufficiently high radio-frequency power of about 10–20 eV. This was enough to break down methane into solid carbon and hydrogen gas [9]. The thermal energy required by these processes was thus further reduced to as low as 300 °C in this work. Then, the C–H bond was dissociated, producing various active groups of carbonaceous species. Methane can be dissociated into a variety of ingredients, including ions, atoms, and active groups (excited state), such as CH_x_ (x = 0–3) [20]. These active groups can undergo a series of free-radical reactions, which can be divided into two sorts: combination reactions of free radicals and migration processes of free radicals. The first ones involve two radicals and form non-free radicals. Then, sufficient energy can aid the accumulation of charged carbonaceous ions and the acceleration of their surface diffusion, making the incorporation of charged carbonaceous ions easier. By means of iterative collision, the active groups increased greatly in number. This prompted the creation of extra free radicals, which often results in chain reactions:

$${\mathrm{CH}}_{4}\to C_{x}H_{y}\to \ldots \to C+2H_{2}$$

By collision and reaction, active groups constantly migrated to the substrate surface and fell on it. Once exceeding a certain threshold, they bonded with each other and then grew into stable groups. This corresponded to the early stage of nucleation of the NGFs. Later, the carbon nuclei combined with each other and stacked into the films.

It was proved that if too high temperatures are reached and too much energy is accumulated, the carbonaceous ions diffuse too fast to grow horizontally [28]. That is, the carbonaceous ions can only stack upwards before new nucleation occurs. The energy provided by PECVD in this work was high enough, making it more likely for the ions to form a stack of nanographite films rather than to grow graphene layer by layer. On the other hand, low substrate temperatures and pressures contrast the migration and diffusion of carbonaceous ions [29]. By slowing down the deposition rate, the possibility of longitudinal growth can be reduced, which is conducive to the growth of nanofilms rather than to the island growth of graphite particles. A glass substrate with smooth surface rather than a catalyzed substrate was a disadvantage to the nucleation of the film and could also slow down film formation. Meanwhile, as affluent energetic H atoms might etch the accreting layers or remove adatoms, the feed gases in this work was methane alone without hydrogen. Thus, carbonaceous species absorbed on the surface would move along the surface quickly. This was also helpful to the low-dimension growth of nanofilms. Meanwhile, a relative low temperature (300 °C) could decrease the nucleation density and slow down nucleation rates, which benefitted the two-dimensional growth of nanographite. This explanation can further prove the Raman result that the carbon films were low-dimension nanograohite films, rather than bulk graphite films. Unfortunately, this is averse to the coverage of the films as well and can account for the unevenness of the films. These factors can well explain the reason why the films were nanographite films [30].

## 4. Conclusions

Low-dimensional carbon nanomaterials of NGFs were synthesized directly on glass substrates under 300 °C by PECVD. NGFs were found to be nanographite films with different thicknesses at the nanoscale. The XPS proved the component and bonding structure to be nanocarbon in sp^2^ bonding form. The Raman spectral characterization verified the configuration of the films to be hexatomic ring networks of carbon atoms. The AFM topography and Raman spectrum of different points demonstrated the nonuniformity of the films on the nanoscale. It was found that the NGFs possessed excellent optic-electronic properties, which is very beneficial for their use as a photoelectrical function material in photoelectric devices, especially as a transparent conductive layer. It was proved that different thicknesses of nanographite films with different morphologies and structures could be obtained by adjusting the parameters during the deposition. In addition, methane was the sole source gas that was used in this work. The absence of a catalyst allowed to obtain a high purity of the films. Furthermore, the complicated transfer process used for optoelectronic devices could be avoided. By directly depositing the films on a glass substrate, quality degradation of the films could be avoided. This work can further promote the development of next-generation electronic or optoelectronic materials, especially for their application in transparent conductive electrode fields.

## Figures and Tables

**Table 1:** The parameters in the deposition of nanographitic films (NGFs). RF: radio frequency.

Vacuum Pressure/Pa	Substrate Temperature/°C	RF Power/W	Gas Flow/Sccm CH_4_:Ar	Deposition Time/Min	Plate Spacing/Cm
50	300	200	2:50	60	5

**Table 2:** Linewidth of D and G peaks and Raman intensity ratios of 2D and G peaks (I_2D_/I_G_). FWHM: full width at half maximum.

Points	1(Red Arrow)	2 (Purple Arrow)	3(Blue Arrow)
FWHM of D/cm^−1^	164	126	134
FWHM of G/cm^−1^	105	80	162
I_2D_/I_G_	0.30	0.32	0.22
